# Association of Dietary Glycemic Index and Glycemic Load With Proatherogenic Factors and Carotid Intima‐Media Thickness in Patients With Metabolic Syndrome

**DOI:** 10.1155/jnme/6289455

**Published:** 2026-06-19

**Authors:** Mehran Rahimlou, Soudabeh Hamedi-Shahraki, Farshad Amirkhizi

**Affiliations:** ^1^ Department of Nutrition, Faculty of Medicine, Zanjan University of Medical Sciences, Zanjan, Iran, zums.ac.ir; ^2^ Department of Epidemiology and Biostatistics, School of Public Health, Zabol University of Medical Sciences, Zabol, Iran, zbmu.ac.ir; ^3^ Department of Nutrition, School of Public Health, Zabol University of Medical Sciences, Zabol, Iran, zbmu.ac.ir

**Keywords:** atherosclerosis, carotid intima-media thickness, glycemic index, glycemic load, inflammation, metabolic syndrome

## Abstract

Metabolic syndrome (MetS) is associated with an increased risk of cardiovascular disease (CVD); however, its etiology—particularly its relationship with dietary factors—remains poorly understood. The study aimed to investigate the relationship between dietary glycemic index (GI) and glycemic load (GL) with carotid intima‐media thickness (cIMT) and proatherogenic inflammatory markers in patients with MetS. This cross‐sectional study was conducted among 185 subjects (80 males and 105 females) aged 20–50 years. Dietary intake was assessed using a validated food frequency questionnaire, and dietary GI and GL were calculated. Main outcomes were cIMT as well as serum levels of malondialdehyde (MDA), high‐sensitivity C‐reactive protein (hs‐CRP), tumor necrosis factor‐alpha (TNF‐α), intercellular adhesion molecule‐1 (ICAM‐1), vascular cell adhesion molecule‐1 (VCAM‐1), and E‐selectin. There was a significant trend indicating that higher GI and GL were associated with elevated serum levels of MDA, TNF‐α, and VCAM‐1. In addition, a significant trend was observed indicating an increase in cIMT across higher tertile categories of both GI and GL. However, dietary GI and GL were not associated with serum levels of hs‐CRP, ICAM‐1, and E‐selectin. Higher dietary GI and GL are associated with increased cIMT, elevated oxidative stress, and proatherogenic inflammatory markers in patients with MetS. These findings suggest that diets high in GI and GL may contribute to the progression of atherosclerosis in this population. Therefore, low‐GI and low‐GL diets may help reduce the risk of CVD and should be considered in dietary recommendations for these patients.

## 1. Introduction

Metabolic syndrome (MetS) is a clinical constellation of interrelated cardiometabolic abnormalities, encompassing central adiposity, impaired fasting glucose, atherogenic dyslipidemia, and elevated blood pressure. This cluster of risk factors synergistically elevates an individual’s predisposition to developing cardiovascular disease (CVD) and type 2 diabetes mellitus [1]. MetS exhibits a rising global prevalence, with current estimates suggesting it affects nearly one‐quarter of the adult population (20%–25%) worldwide [2]. This upward trend is largely attributable to the escalating incidence of obesity and the adoption of increasingly sedentary patterns of living [2]. In Iran, the syndrome’s prevalence is notably elevated, with certain studies documenting rates reaching 30% within specific demographic groups, a finding that highlights the critical necessity for implementing effective preventive public health measures [3]. Dietary patterns represent a crucial modifiable risk factor for MetS, exerting a significant influence on its development through their impact on underlying metabolic processes and inflammatory pathways [4, 5].

The glycemic index (GI) and glycemic load (GL) are critical metrics for evaluating the quality and quantity of dietary carbohydrates, respectively [6]. The GI quantifies the postprandial blood glucose response elicited by a carbohydrate‐rich food, standardized against a reference food such as pure glucose or white bread. In contrast, the GL provides a more integrated measure by accounting for both the GI of a food and the absolute quantity of carbohydrates ingested in a typical serving [6]. High‐GI and high‐GL diets, characterized by rapid glucose spikes, have been implicated in exacerbating oxidative stress [7, 8] and systemic inflammation [9], hallmarks of MetS and precursors to atherosclerosis. Carotid intima‐media thickness (cIMT), a validated marker of subclinical atherosclerosis, is strongly associated with metabolic disturbances and serves as an early indicator of CVD risk [10]. Additionally, proatherogenic inflammatory markers such as tumor necrosis factor‐alpha (TNF‐α) and vascular cell adhesion molecule‐1 (VCAM‐1), along with oxidative stress markers including malondialdehyde (MDA), are elevated in MetS and are believed to mediate the progression of atherosclerosis [11–13]. However, the role of dietary factors in elevating these markers in patients with MetS remains not fully understood.

While a substantial body of research has linked high‐GI/GL diets to an elevated risk of CVD within the general populace [14–16], the precise pathophysiological mechanisms underlying this association remain inadequately characterized. Furthermore, the extant literature is marked by considerable inconsistency, as several prominent investigations have reported no significant correlation between the consumption of high GI/GL diets and cardiovascular outcomes [17, 18].

In the Iranian population, dietary carbohydrates constitute nearly two‐thirds of total daily caloric intake [19]. Refined grain products, predominantly bread and white rice, are the primary sources, representing 34.2% and 14.8% of total carbohydrate consumption, respectively [20]. Notably, a substantial proportion of staple carbohydrate‐based foods within the traditional Iranian diet—such as various breads, rice, potatoes, and processed snacks—exhibit high GI values. The intersection of these customary dietary practices with the nation’s elevated prevalence of MetS, coupled with the persistent scientific requirement to clarify the pathophysiological pathways by which high‐GI/GL diets potentiate CVD risk in MetS, underscores the critical necessity for focused research on the role of dietary GIs in promoting both atherogenesis and chronic inflammatory pathways.

This study aimed to investigate the associations between dietary GI and GL with cIMT and a comprehensive panel of proatherogenic markers—including oxidative stress (MDA), inflammatory cytokines (TNF‐α), cellular adhesion molecules (VCAM‐1, ICAM‐1, E‐selectin), and systemic inflammation (hs‐CRP)—in Iranian adults with MetS.

## 2. Materials and Methods

### 2.1. Study Setting and Participants

This research employed a cross‐sectional design to investigate a cohort of 185 individuals diagnosed with MetS, ranging from 20 to 50 years of age. The participants were recruited from health centers in Tehran, Iran.

The requisite number of subjects was derived from a power analysis using serum E‐selectin concentration—the designated primary endpoint—with reference to previously published data [21]. Employing a standard formula for cross‐sectional research, and given a standard deviation of 21, a margin of error (*d*) of 3, and an alpha value of 0.05, the computation yielded a minimum sample size of 185 participants.

A diagnosis of MetS was assigned to participants according to established international criteria [22]. This required the manifestation of a minimum of three abnormal clinical findings, comprising elevated fasting blood glucose (FBG ≥ 100 mg/dL); hypertriglyceridemia (TG ≥ 150 mg/dL); hypertension (systolic/diastolic BP ≥ 130/85 mmHg); increased waist circumference (WC ≥ 95 cm, applied uniformly across sexes); or reduced high‐density lipoprotein cholesterol (HDL‐C < 40 mg/dL for males and < 50 mg/dL for females). The threshold for abdominal obesity, defined by waist circumference, was established in accordance with population‐specific criteria issued by the Iranian National Committee of Obesity [23].

Eligibility for this research was confined to individuals aged 20–50 years, irrespective of gender, who provided informed consent. The exclusion criteria encompassed a history of clinically diagnosed comorbidities, including CVD, renal failure, diabetes mellitus, hepatic impairment, neoplasms, thyroid disorders (hypothyroidism or hyperthyroidism), cholelithiasis, biliary obstruction, inflammatory conditions, or recent surgical procedures. Further grounds for exclusion were the current or recent (within 3 months preceding the study) use of omega‐3 fatty acids, antioxidant supplements (e.g., vitamin C, vitamin E, or selenium), or pharmaceutical agents for lipid reduction or hypertension management. Finally, prospective female participants were deemed ineligible if they were pregnant, lactating, or postmenopausal.

### 2.2. Ethical Approval

Prior to data acquisition, all participants received a comprehensive explanation of the study’s aims and procedures, after which written informed consent was obtained from each individual. The research protocol received formal approval from the Ethics Committee at Zabol University of Medical Sciences (approval code: IR.ZBMU.REC.1404.076).

### 2.3. Dietary Intake and Physical Activity Assessment

A certified dietitian assessed habitual dietary intake through a 168‐item, semiquantitative food frequency questionnaire (FFQ). The instrument’s validity and reliability have been previously confirmed within this population [24]. Dietary assessment was conducted using a standardized FFQ, where participants self‐reported their habitual intake over the preceding year. For each food item, they indicated the frequency of consumption (daily, weekly, monthly, or yearly) and specified portion sizes. These reported serving sizes were subsequently converted into metric units (grams and milliliters) based on standardized household measures. The resulting quantitative data were analyzed for nutrient composition using Nutritionist IV software.

Physical activity levels were evaluated using the short form of the International Physical Activity Questionnaire (IPAQ), which was administered via an interviewer‐led process. This instrument comprises seven items designed to quantify the frequency and duration of vigorous‐intensity and moderate‐intensity activities, walking, and sedentary time spent sitting over a 7‐day recall period. The cumulative scores for these activities are calculated and expressed as metabolic equivalent of task (MET) minutes per week [25]. The validity of the Persian‐language version of the short‐form IPAQ has been established in prior research, demonstrating strong internal consistency (Cronbach’s *α* = 0.7) and excellent temporal reliability (test–retest coefficient = 0.9) [26].

### 2.4. Anthropometric Measures and Body Composition Assessment

Body composition analysis was performed via bioelectrical impedance analysis (BIA) with an InBody 770 device (InBody Co., Seoul, Korea). All measurements were obtained in the morning between 8:00 and 9:00 a.m., following a 12 h overnight fast. In adherence to the manufacturer’s protocol, participants were instructed to remove heavy outer garments, footwear, and all metallic accessories such as jewelry, watches, and belts. The non‐invasive procedure requires approximately 20 s to complete, generating estimates of both total fat mass and visceral adipose tissue percentage.

Anthropometric measurements were collected using calibrated instruments. Body weight was determined to the nearest 0.1 kg using a digital Seca scale (Seca, Hamburg, Germany), and standing height was assessed to the nearest 0.5 cm with a Seca stadiometer. Body mass index (BMI) was derived from these measures using the standard formula: weight in kilograms divided by the square of height in meters (kg/m^2^).

WC was ascertained with a non‐elastic tape measure precise to 1.0 cm. With the participant in a standing position, the measurement was taken at the end of a normal expiration at the midpoint between the inferior margin of the last rib and the iliac crest.

### 2.5. GI and GL Calculation

GI values, which quantify the postprandial glycemic response of carbohydrate‐rich foods as a percentage relative to a glucose reference, were obtained from two primary sources: the International Glycemic Index Table (2008) by Atkinson and colleagues [27], and supplementary datasets published by the University of Sydney [28].

Simple carbohydrates, which lead to a quick spike in blood glucose, have a higher GI. In contrast, high‐fiber foods, which slow the absorption of glucose, typically have a lower GI [29].

For traditional Iranian food items absent from international databases, corresponding GI and GL values were ascertained from nationally published nutritional resources [30]. In instances where a specific GI value remained unavailable, an equivalent value from a nutritionally analogous food item was applied. The overall dietary GI and GL for each participant were subsequently computed according to established methodologies, utilizing the standard formulas [31, 32]:

(1) Dietary GI=Carbohydrate content of each food item×number of servings/day×GITotal daily carbohydrate intake,Dietary GL=Carbohydrate content of each food item×number of servings/day×GI.

The GI values were standardized using white bread as the reference food (GI = 100). For food items with multiple published GI values, a mean value was computed from the available data and employed in the subsequent analysis.

### 2.6. cIMT Measurement

Assessment of common cIMT was performed by a certified radiologist utilizing B‐mode ultrasonography (Accuvix V10, Medison). In accordance with established methodology [33], imaging was conducted with a 7 MHz linear transducer following a five‐minute rest period in the supine position. The target segment was the far wall of the common carotid artery, approximately 2 cm proximal to the bifurcation. Participants were directed to gently rotate their head to each side to facilitate bilateral access. The cIMT was measured on both the right and left carotid arteries, with the maximum value from each side documented. The mean of these two maximum values was subsequently calculated and used as the final cIMT for analysis.

To ensure measurement reliability, all scans were analyzed by a single experienced reader. Intra‐reader reliability was assessed by having the same reader re‐analyze a random subset of 20 scans after a 2‐week interval, with the reader blinded to the initial results. The repeatability was quantified using the intraclass correlation coefficient (ICC) for absolute agreement. The reliability of the cIMT measurements was good, with an intra‐reader ICC of 0.82 (95% CI: 0.78–0.86).

### 2.7. Laboratory Measurements

After an overnight fast of at least 10 h, a 10 mL venous blood sample was drawn from the antecubital fossa of each subject. The samples were subsequently centrifuged at 4°C for 10 min at 3000 revolutions per minute to separate the serum component. The resulting serum aliquots were immediately frozen at −80°C for preservation until batch analysis could be performed.

The concentration of MDA in serum was quantified using the thiobarbituric acid reactive substances (TBARS) assay, in accordance with the methodology established by Uchiyama and Mihara [34]. This assay quantifies MDA concentration by measuring the absorbance of a pink‐colored chromogen at 532 nm, which is generated by the specific reaction between MDA and thiobarbituric acid. The intra‐ and interassay coefficients of variation (CVs) for this biomarker were 7.8% and 9.1%, respectively.

Serum analysis of inflammatory biomarkers was conducted using standardized commercial assays. hs‐CRP levels were assessed via an immunoturbidimetric technique (Pars Azmoon Co., Tehran, Iran). TNF‐α concentrations were determined with enzyme‐linked immunosorbent assay (ELISA) kits sourced from Bioassay Technology Laboratory (Shanghai, China), in strict adherence to the manufacturer’s instructions. The intra‐ and inter‐assay CVs were as follows: for hs‐CRP, 8.7% and 6.5%; for TNF‐α, 7.7% and 5.4%, respectively. Similarly, concentrations of ICAM‐1, VCAM‐1, and E‐selectin were measured using specific ELISA kits (Platinum series; eBioscience, Vienna, Austria) according to the protocols provided by the supplier. The intra‐ and interassay CVs were 7.4% and 5.6% for ICAM‐1, 9.3% and 4.9% for VCAM‐1, and 7.3% and 5.8% for E‐selectin, respectively.

### 2.8. Statistical Analysis

First, energy‐adjusted GI and GL were calculated using the residual method [35]. Participants were then categorized into tertiles based on these adjusted values. Baseline characteristics are presented as mean ± standard deviation for continuous variables and as frequency (percentage) for categorical variables. The normality of data distribution was assessed using the Kolmogorov–Smirnov test. Intertertile differences were evaluated using analysis of variance (ANOVA) for continuous variables and the chi‐square test for categorical variables. The relationships between dietary GI/GL and outcomes—including cIMT, oxidative stress markers, endothelial dysfunction indicators, and systemic inflammatory biomarkers—were examined using multiple linear regression. Analyses were performed using both unadjusted (crude) models and multivariable models adjusted for potential confounders, including age, sex, smoking status, physical activity, and BMI.

## 3. Results

Of the 185 participants enrolled in the study, 43.2% were male and 56.8% were female. The mean age of the cohort was 39.0 ± 5.5 years, with a mean BMI of 31.5 ± 2.1 kg/m^2^. Median values for GI across tertiles ranged from 34.5 to 109.8, while GL values ranged from 82.7 to 305.2. The primary dietary sources of GL were bread (38.2%), rice (23.7%), fruits (14.7%), and sweets (10.7%). A comprehensive summary of participant demographic characteristics, dietary intake patterns, and anthropometric measurements, stratified by GI and GL tertiles, is provided in Table 1.

**TABLE 1:** Demographic characteristics, dietary intakes, and anthropometric measures of the participants across tertiles of GI and GL.

Variables	GI tertiles^a^			*p* ^c^	GL tertiles^b^			*p* ^d^
	T1 (*n* = 62)	T2 (*n* = 62)	T3 (*n* = 61)		T1 (*n* = 62)	T2 (*n* = 62)	T3 (*n* = 61)	
Sex, *n* (%)								
Male	27 (43.5)	26 (41.9)	27 (44.3)	0.965	27 (43.5)	24 (38.7)	29 (47.5)	0.612
Female	35 (56.5)	36 (58.1)	34 (55.7)		35 (56.5)	38 (61.3)	32 (52.5)	
Age (years)	39.6 ± 6.1	38.5 ± 5.5	38.9 ± 5.0	0.543	39.2 ± 6.2	39.9 ± 5.2	38.0 ± 5.0	0.139
Weight (kg)	91.3 ± 12.2	89.8 ± 12.1	90.8 ± 11.1	0.761	90.6 ± 11.1	89.8 ± 11.7	91.5 ± 12.4	0.737
BMI (kg/m^2)^	31.3 ± 2.2	31.3 ± 2.1	31.9 ± 1.9	0.129	31.5 ± 2.0	31.6 ± 2.0	31.4 ± 2.0	0.961
Fat mass (%)	38.8 ± 8.0	36.5 ± 8.4	40.3 ± 7.5	0.128	39.0 ± 7.5	38.6 ± 8.2	37.9 ± 8.5	0.740
Visceral fat (%)	14.0 ± 4.2	12.6 ± 4.2	13.9 ± 3.8	0.103	13.8 ± 4.1	13.9 ± 4.3	12.9 ± 3.8	0.361
Current smokers, *n* (%)	11 (17.7)	9 (14.5)	16 (26.2)	0.238	13 (21.0)	10 (16.1)	13 (21.3)	0.718
Physical activity (METs‐hr/wk)	28.9 ± 6.9	30.4 ± 6.3	30.6 ± 6.6	0.296	28.5 ± 6.7	30.1 ± 6.5	31.2 ± 6.5	0.072
Dietary intakes^c^								
Energy (kcal/day)	2443 ± 697	2330 ± 730	2489 ± 725	0.495	2355 ± 655	2441 ± 752	2467 ± 746	0.714
Carbohydrate (g/day)	351 ± 90	376 ± 98	409 ± 110	0.013	356 ± 95	374 ± 100	407 ± 105	0.032
Protein (g/day)	72.5 ± 19.5	70.5 ± 19.8	77.9 ± 20.3	0.137	71.1 ± 18.9	71.7 ± 20.7	78.0 ± 20.0	0.147
Fats (g/day)	70.0 ± 23.6	67.7 ± 26.1	68.3 ± 22.1	0.878	65.8 ± 22.2	68.6 ± 22.5	71.5 ± 26.8	0.475
Whole grains (g/d)	22.7 ± 9.7	28.5 ± 12.5	26.2 ± 10.3	0.232	28.6 ± 10.4	30.5 ± 11.7	31.9 ± 12.6	0.435
Refined grains (g/d)	382 ± 132	486 ± 148	536 ± 139	< 0.001	393 ± 126	508 ± 132	612 ± 182	< 0.001
Fruits (g/d)	321 ± 95	379 ± 121	498 ± 171	0.002	375 ± 116	407 ± 134	571 ± 167	< 0.001
Vegetables (g/d)	457 ± 103	371 ± 124	318 ± 114	0.001	421 ± 167	397 ± 113	317 ± 121	0.005
Legumes and nuts (g/d)	78.7 ± 12.6	68.6 ± 14.2	64.9 ± 15.4	0.574	81.5 ± 14.8	77.9 ± 15.7	76.4 ± 16.8	0.438
Meats (g/d)	148 ± 71	152 ± 65	161 ± 58	0.632	138 ± 82	147 ± 71	141 ± 62	0.497
Dairy products (g/d)	371 ± 114	398 ± 127	369 ± 107	0.873	403 ± 135	378 ± 114	347 ± 131	0.458
Sweets (g/d)	112 ± 42	141 ± 49	173 ± 64	< 0.001	124 ± 53	153 ± 74	196 ± 87	< 0.001

*Note:* Data are reported as mean ± standard deviation unless indicated otherwise.

Abbreviations: BMI, body mass index; GI, glycemic index; GL, glycemic load.

^a^Tertile cut points of GI are as follows: first, < 60.07; second, 60.08 to 65.81; third, > 65.81.

^b^Tertile cut points of GL are as follows: first, < 131.92; second, 131.93 to 195.4; third, > 195.4.

^c^Energy intake is adjusted for age and sex; all other dietary intake items are adjusted for age, sex, and energy intake.

^d^Determined by one‐way ANOVA test or Pearson chi‐square test for continuous or categorical variables, respectively.

The analysis revealed no statistically significant variations in mean age, BMI, physical activity levels, body fat mass, or visceral adipose tissue percentage across the tertiles of dietary GI and GL. Similarly, the distribution of sex and prevalence of current smoking did not differ significantly among the tertile groups. When compared to participants in the lowest tertile, individuals in the upper tertiles of both GI and GL demonstrated significantly higher consumption of carbohydrates, refined grains, fruits, and sugar‐sweetened foods. Conversely, vegetable intake was notably lower among participants in the highest tertile relative to those in the lowest tertile. No significant differences were observed across tertiles for total energy intake or the consumption of protein, fats, whole grains, legumes, nuts, meat, or dairy products, as detailed in Table 1.

Oxidative stress, endothelial function, and systemic inflammatory biomarkers stratified by dietary GI and GL tertiles are presented in Table 2. The data reveal a significant positive trend, whereby ascending tertiles of both GI and GL were associated with progressively higher circulating concentrations of MDA, TNF‐α, and VCAM‐1. A similar significant trend was observed for cIMT, which increased across higher tertile categories of dietary GI and GL. However, dietary GI and GL were not associated with serum levels of hs‐CRP, ICAM‐1, and E‐selectin.

**TABLE 2:** Oxidative stress, endothelial, and systemic inflammatory markers in the participants across tertiles of dietary GI and GL.

Variables	GI tertiles^a^			*p* trend^c^	GL tertiles^b^			*p* trend^c^
	T1 (*n* = 62)	T2 (*n* = 62)	T3 (*n* = 61)		T1 (*n* = 62)	T2 (*n* = 62)	T3 (*n* = 61)	
MDA (nmol/mL)	1.72 ± 0.39	1.92 ± 0.46	2.10 ± 0.51	< 0.001	1.73 ± 0.43	1.93 ± 0.44	2.08 ± 0.50	< 0.001
hs‐CRP (mg/L	4.77 ± 2.77	4.77 ± 3.23	5.19 ± 3.09	0.440	5.04 ± 2.94	4.81 ± 2.72	4.87 ± 3.44	0.764
TNF‐α (pg/mL)	23.76 ± 5.29	25.83 ± 4.18	26.36 ± 4.42	0.002	24.20 ± 5.21	24.81 ± 4.22	26.95 ± 4.44	0.001
ICAM‐1 (ng/mL)	373.4 ± 74.6	381.0 ± 84.3	379.7 ± 87.5	0.671	359.4 ± 72.3	390.5 ± 92.1	384.2 ± 78.0	0.092
VCAM‐1 (ng/mL)	688.9 ± 140.3	755.4 ± 132.6	754.5 ± 134.3	0.008	678.4 ± 135.2	766.7 ± 126.3	753.6 ± 139.4	0.002
E‐selectin (ng/mL)	27.43 ± 7.94	29.45 ± 10.35	28.57 ± 7.75	0.743	29.09 ± 8.94	27.99 ± 8.17	28.37 ± 9.24	0.648
cIMIT (mm)	0.69 ± 0.12	0.73 ± 0.11	0.75 ± 0.13	0.008	0.69 ± 0.12	0.72 ± 0.12	0.76 ± 0.12	0.004

*Note:* Data are illustrated as mean ± standard deviation.

Abbreviations: cIMT, carotid intima‐media thickness; GI, glycemic index; GL, glycemic load; hs‐CRP, high‐sensitivity C‐reactive protein; ICAM‐1, intercellular adhesion molecule 1; MDA, malondialdehyde; TNF‐α, tumor necrosis factor‐α; VCAM‐1, vascular cell adhesion molecule‐1.

^a^Tertile cut points of glycemic index are as follows: first, < 60.07; second, 60.08 to 65.81; third, > 65.81.

^b^Tertile cut points of glycemic load are as follows: first, < 131.92; second, 131.93 to 195.4; third, > 195.4.

^c^Determined by one‐way ANOVA test.

Table 3 presents the findings from multiple linear regression analyses evaluating the associations between dietary GI and GL (independent variables) and several dependent variables, including oxidative stress markers, endothelial and inflammatory biomarkers, and cIMT. The regression analysis demonstrated that serum concentrations of MDA, TNF‐α, VCAM‐1, and cIMT were significantly dependent on dietary GI and GL in the study population. In the unadjusted model, a positive association was observed between both GI and GL scores and the levels of these biomarkers and cIMT. These positive relationships persisted after adjustment for potential confounding variables, namely age, sex, smoking status, physical activity, and BMI.

**TABLE 3:** Beta (*β*) and 95% confidence interval for oxidative stress, endothelial, and systemic inflammatory markers across tertiles of dietary GI and GL.

Variables	GI tertiles^a^			*p* trend^d^	GL tertiles^b^			*p* trend^d^
	T1 (*n* = 62)	T2 (*n* = 62)	T3 (*n* = 61)		T1 (*n* = 62)	T2 (*n* = 62)	T3 (*n* = 61)	
MDA								
Crude model	Ref.	0.21 (0.05, 0.37)	0.38 (0.22, 0.55)	< 0.001	Ref.	0.21 (0.05, 0.37)	0.36 (0.19, 0.52)	< 0.001
Adjusted model^c^	Ref.	0.20 (0.04, 0.36)	0.40 (0.24, 0.56)	< 0.001	Ref.	0.19 (0.03, 0.36)	0.36 (0.19, 0.52)	< 0.001
hs‐CRP								
Crude model	Ref.	−0.01 (0.99, 1.07)	0.42 (−0.66, 1.07)	0.440	Ref.	−0.23 (−1.31, 0.85)	−0.16 (−1.25, 0.92)	0.763
Adjusted model^c^	Ref.	−0.04 (−1.13, 1.05)	0.33 (−0.77, 1.44)	0.554	Ref.	−0.35 (−1.45, 0.74)	−0.28 (−1.39, 0.82)	0.612
TNF‐α								
Crude model	Ref.	2.07 (0.42, 3.72)	2.60 (0.95, 4.26)	0.002	Ref.	0.61 (−1.04, 2.25)	2.75 (1.09, 4.40)	0.001
Adjusted model^c^	Ref.	2.06 (0.38, 3.74)	2.59 (0.89, 4.28)	0.003	Ref.	0.52 (−1.16, 2.21)	2.66 (0.96, 4.36)	0.002
ICAM‐1								
Crude model	Ref.	7.60 (−21.58, 36.77)	6.32 (−22.98, 35.61)	0.669	Ref.	31.03 (2.22, 59.83)	24.80 (−4.12, 53.72)	0.092
Adjusted model^c^	Ref.	7.00 (−22.50, 36.49)	4.33 (−25.53, 34.20)	0.771	Ref.	29.39 (0.05, 58.74)	24.30 (−5.27, 53.87)	0.107
VCAM‐1								
Crude model	Ref.	66.5 (18.4, 114.6)	65.6 (17.2, 113.9)	0.008	Ref.	88.3 (40.9, 135.7)	75.2 (27.6, 122.7)	0.002
Adjusted model^c^	Ref.	67.7 (18.9, 116.5)	62.8 (13.4, 112.3)	0.013	Ref.	90.5 (40.9, 135.7)	77.5 (28.9, 126.2)	0.002
E‐Selectin								
Crude model	Ref.	2.02 (−1.08, 5.13)	1.14 (−1.98, 4.25)	0.470	Ref.	−1.10 (−4.22, 2.01)	−0.73 (−3.85, 2.40)	0.645
Adjusted model^c^	Ref.	2.31 (−0.81, 5.43)	0.98 (−2.18, 4.15)	0.531	Ref.	−1.10 (−4.26, 2.06)	−0.56 (−3.75, 2.62)	0.726
cIMIT								
Crude model	Ref.	0.04 (−0.01, 0.08)	0.06 (0.02, 0.10)	0.008	Ref.	0.03 (−0.01, 0.08)	0.06 (0.02, 0.11)	0.003
Adjusted model^c^	Ref.	0.03 (−0.01, 0.07)	0.05 (0.012, 0.10)	0.014	Ref.	0.03 (−0.01, 0.07)	0.06 (0.02, 0.10)	0.007

Abbreviations: cIMT, carotid intima‐media thickness; GI, glycemic index; GL, glycemic load; hs‐CRP, high‐sensitivity C‐reactive protein; ICAM‐1, intercellular adhesion molecule 1; MDA, malondialdehyde; TNF‐α, tumor necrosis factor‐α; VCAM‐1, vascular cell adhesion molecule‐1.

^a^Tertile cut points of glycemic index are as follows: first, < 60.07; second, 60.08 to 65.81; third, > 65.81.

^b^Tertile cut points of glycemic load are as follows: first, < 131.92; second, 131.93 to 195.4; third, > 195.4.

^c^Adjusted for age, sex, smoking status, physical activity level, and BMI.

^d^Determined by linear regression. *p* < 0.05 was considered statistically significant.

## 4. Discussion

This cross‐sectional investigation of 185 individuals with MetS revealed that elevated dietary GI and GL were significantly associated with greater cIMT and higher circulating concentrations of MDA, TNF‐α, and VCAM‐1. These relationships persisted after controlling for potential confounding variables, including age, sex, smoking status, physical activity, and BMI. Conversely, no statistically significant associations were detected for serum concentrations of high‐hs‐CRP, ICAM‐1, or E‐selectin. Collectively, these results underscore the potential contribution of high‐GI and high‐GL dietary patterns to the advancement of pro‐atherogenic inflammation and subclinical atherosclerosis in patients with MetS.

The findings of this study are consistent with, and expand upon, a growing body of literature highlighting the proinflammatory and atherogenic potential of high‐GI/GL diets. Several observational and clinical studies have reported similar associations between carbohydrate quality and markers of systemic and endothelial inflammation. For example, in a cross‐sectional analysis of patients with type 2 diabetes, Chiavaroli et al. found that dietary GI was positively associated with cIMT, suggesting a dietary influence on early atherosclerotic changes [36]. Similarly, Zhang et al. demonstrated that chronic hyperglycemia serves as a bridge linking MetS to CVD through inflammation‐mediated pathways [37]. Elevated postprandial glucose excursions, which are common in high‐GI diets, have been shown to induce endothelial dysfunction and upregulate the expression of adhesion molecules such as VCAM‐1 and ICAM‐1, facilitating leukocyte adhesion and migration [38].

Biologically, the mechanisms underpinning our observations likely involve the interplay between glycemic fluctuations, oxidative stress, and inflammatory signaling cascades [39, 40]. High‐GI meals induce rapid glucose absorption and hyperinsulinemia, which in turn stimulate the production of ROS, particularly within vascular endothelial cells [41]. MDA, a by‐product of lipid peroxidation, serves as a surrogate marker for oxidative damage and was significantly elevated in our high‐GI/GL groups. ROS can activate nuclear factor‐κB (NF‐κB) signaling, promoting the transcription of proinflammatory cytokines, including TNF‐α, and adhesion molecules like VCAM‐1. This sequence of events contributes to endothelial dysfunction and progression of subclinical atherosclerosis, evidenced by increased cIMT [42, 43].

The lack of a significant correlation with hs‐CRP observed in this analysis is notable and may be attributed to the complex, multifactorial nature of its physiological regulation. Although certain epidemiological investigations have proposed a potential link between dietary carbohydrate quality and CRP levels, the reported findings across the literature remain inconsistent. For instance, several dietary intervention trials have reported a decrease in CRP concentrations following reduced consumption of high‐GI/GL foods; however, this effect has not been universally observed across studies [39, 44]. This inconsistency may be attributable to variations in participants’ baseline inflammatory status, the specific nutrient profiles of the intervention diets, or differences in trial duration. The absence of significant associations with E‐selectin and ICAM‐1 in the present analysis suggests that susceptibility to dietary glycemic exposure may vary across specific endothelial biomarkers. This may indicate that not all markers of endothelial activation are equally responsive to short‐term nutritional influences and that observable changes in certain pathways might necessitate interventions of extended duration. Hyperglycemia stimulates the production of proinflammatory cytokines such as TNF‐α, which activates NF‐κB pathways, upregulating adhesion molecules like VCAM‐1 [45]. VCAM‐1 facilitates monocyte adhesion to the vascular endothelium, a critical step in atherogenesis.

The absence of an association with hs‐CRP, ICAM‐1, and E‐selectin in our study suggests that high‐GI/GL diets may preferentially influence specific inflammatory pathways, particularly those involving TNF‐α and VCAM‐1, in patients with MetS. This selective effect warrants further investigation to elucidate the precise molecular mechanisms involved.

From a clinical perspective, these findings underscore the importance of considering carbohydrate quality—not just quantity—in dietary recommendations for patients with MetS [46]. While total carbohydrate intake did not significantly differ across GI and GL tertiles, the increased consumption of refined grains and sweets among individuals with higher GI/GL scores suggests that food choices, rather than macronutrient balance alone, may drive adverse outcomes [47]. Furthermore, we hypothesize that the synergy between diet‐induced postprandial dysmetabolism—resulting from a refined‐carbohydrate diet—and adipose tissue‐derived inflammation creates a uniquely potent environment for sustained NF‐κB activation and vascular endothelial dysfunction, ultimately accelerating cIMT progression.

These findings have significant implications for dietary recommendations and public health strategies. Substituting high‐GI foods with low‐GI alternatives—such as legumes, whole grains, and fiber‐rich vegetables—may help reduce postprandial glucose spikes, decrease oxidative and inflammatory stress, and potentially slow the progression of atherosclerosis in at‐risk populations. However, the findings of this study are specifically applicable to young and middle‐aged adults with MetS who are free from major concurrent conditions such as diabetes and CVD; they should not be extrapolated to older populations with MetS or to individuals with significant metabolic complications.

Our study has several strengths that enhance the validity of its findings. These include a relatively large sample size, rigorous inclusion and exclusion criteria to minimize confounding by comorbidities or medications, and comprehensive assessments of dietary intake, anthropometrics, and inflammatory markers. The use of validated questionnaires and standardized protocols further increases confidence in the accuracy of our measurements. However, certain limitations must be acknowledged. First, the cross‐sectional design of the study restricts our ability to establish causality. Although we observed significant associations between dietary GI/GL and inflammation/cIMT, longitudinal or interventional studies are needed to confirm these findings and clarify temporal relationships. Second, dietary intake was self‐reported using an FFQ, which is subject to recall bias and potential under‐ or over‐reporting, although the FFQ employed was previously validated in similar populations. Third, residual confounding cannot be entirely excluded despite multivariable adjustments. Factors such as genetic predisposition, gut microbiota composition, or unmeasured dietary variables (e.g., micronutrient status) may have influenced the results. Fourth, our sample was recruited from a single regional health center in Iran. The dietary patterns, socioeconomic factors, and health behaviors in this region may not fully represent the broader Iranian population or other ethnic and geographic groups within the country. Therefore, caution is advised against over‐extrapolating the findings. Fifth, although the FFQ was designed to capture habitual intake, its coverage of traditional and specialty Iranian foods may be incomplete. Finally, the glycemic response to staple Iranian foods—such as specific rice varieties and traditional breads—may be influenced by local cooking methods, preparation techniques, and ingredient composition. Consequently, standardized GI values may not accurately reflect the physiological responses in our population, representing a potential source of measurement error.

In conclusion, our findings indicate that high‐GI/GL diets show independent associations with elevated oxidative stress, increased endothelial/inflammatory markers, and greater cIMT in adults with MetS. These results support the hypothesis that high‐GI/GL diets are associated with vascular inflammation and early atherogenesis. Clinically, dietary interventions targeting GI/GL reduction may represent an effective approach for CVD risk mitigation in this high‐risk population. Future research should employ prospective designs and randomized controlled trials to confirm these associations, investigate mechanistic pathways, and assess long‐term cardiovascular outcomes of GI/GL modification in patients with MetS.

## Author Contributions

Mehran Rahimlou and Farshad Amirkhizi prepared the first draft of the manuscript and coordinated in the interpretation of the data. Soudabeh Hamedi‐Shahraki performed the statistical analysis and takes responsibility for the integrity of the data and the accuracy of the data analysis. Farshad Amirkhizi was involved in the study design, interpretation of the data, supervised data collection, and edited the manuscript.

## Funding

This work was supported by Zabol University of Medical Sciences, 10.13039/501100010802.

## Disclosure

All authors critically reviewed the manuscript and approved the final submitted draft of this manuscript.

## Ethics Statement

All experiments were performed in accordance with the Declaration of Helsinki and Zabol University of Medical Sciences’ ethical guidelines and regulations. The research protocol was approved by the Zabol University of Medical Sciences (approval code: IR.ZBMU.REC.1404.076). All participants signed a written informed consent before participating in the study.

## Conflicts of Interest

The authors declare no conflicts of interest.

## Data Availability

Data will be available on request from the corresponding author.
